# Supplementary material for: Investigating the effect of dependence between conditions with Bayesian Linear Mixed Models for motif activity analysis
Source: PLoS One. 2020 May 1;15(5):e0231824. doi: 10.1371/journal.pone.0231824 (PMC7194367; doi:10.1371/journal.pone.0231824)
Supplement: S1 Fig — Data was generated over G = 978 informative genes and T = 623 motif scores and 100 repetitions, with structured ΣC. The results for Bayesian Linear Mixed Model and Ridge Regression (RIDGE, in blue) are equal when Bayesian Linear Mixed Model is limited to VC = σ2 IC and ΣC = δ IC (BLMM_id, in red). The Pearson correlation values are computed between the generated and predicted posterior motif influence ω^T,C, and separated by method used to compute them. Data is generated with (i) independent samples, VC = IC, (ii) unrestricted correlation between samples, (iii) 50% correlated data, where the samples cluster in many (k=12C) sample groups, (iv) highly correlated data, by generating a covariance matrix with k = 2 completely correlated sample groups, with C the number of conditions. (PDF) [file pone.0231824.s001.pdf]

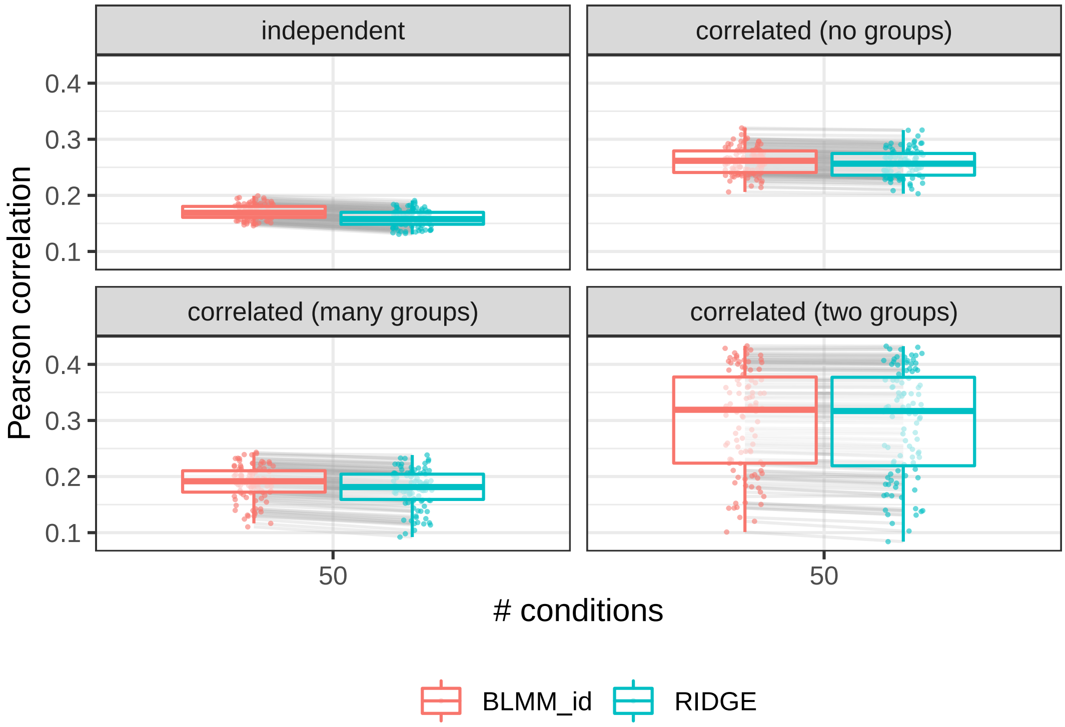

Figure S1: **Ridge Regression is a special case of Bayesian Linear Mixed Model, limiting the estimated covariance and noise to be independent.** Data was generated over $G = 978$ informative genes and $T = 623$ motif scores and 100 repetitions, with structured $\mathbf{\Sigma}_C$. The results for Bayesian Linear Mixed Model and Ridge Regression (RIDGE, in blue) are equal when Bayesian Linear Mixed Model is limited to $\mathbf{V}_C = \sigma^2\mathbf{I}_C$ and $\mathbf{\Sigma}_C = \delta\mathbf{I}_C$ (BLMM_id, in red). The Pearson correlation values are computed between the generated and predicted posterior motif influence $\hat{\omega}_{T,C}$, and separated by method used to compute them. Data is generated with (i) independent samples, $\mathbf{V}_C = \mathbf{I}_C$, (ii) unrestricted correlation between samples, (iii) 50% correlated data, where the samples cluster in many ($k = \frac{1}{2}C$) sample groups, (iv) highly correlated data, by generating a covariance matrix with $k = 2$ completely correlated sample groups, with C the number of conditions.
