# Supplementary material for: Investigating the effect of dependence between conditions with Bayesian Linear Mixed Models for motif activity analysis
Source: PLoS One. 2020 May 1;15(5):e0231824. doi: 10.1371/journal.pone.0231824 (PMC7194367; doi:10.1371/journal.pone.0231824)
Supplement: S2 Fig — Results of simulation study analogously presented as in Simulation with Spearman’s rank correlation values. (PDF) [file pone.0231824.s002.pdf]

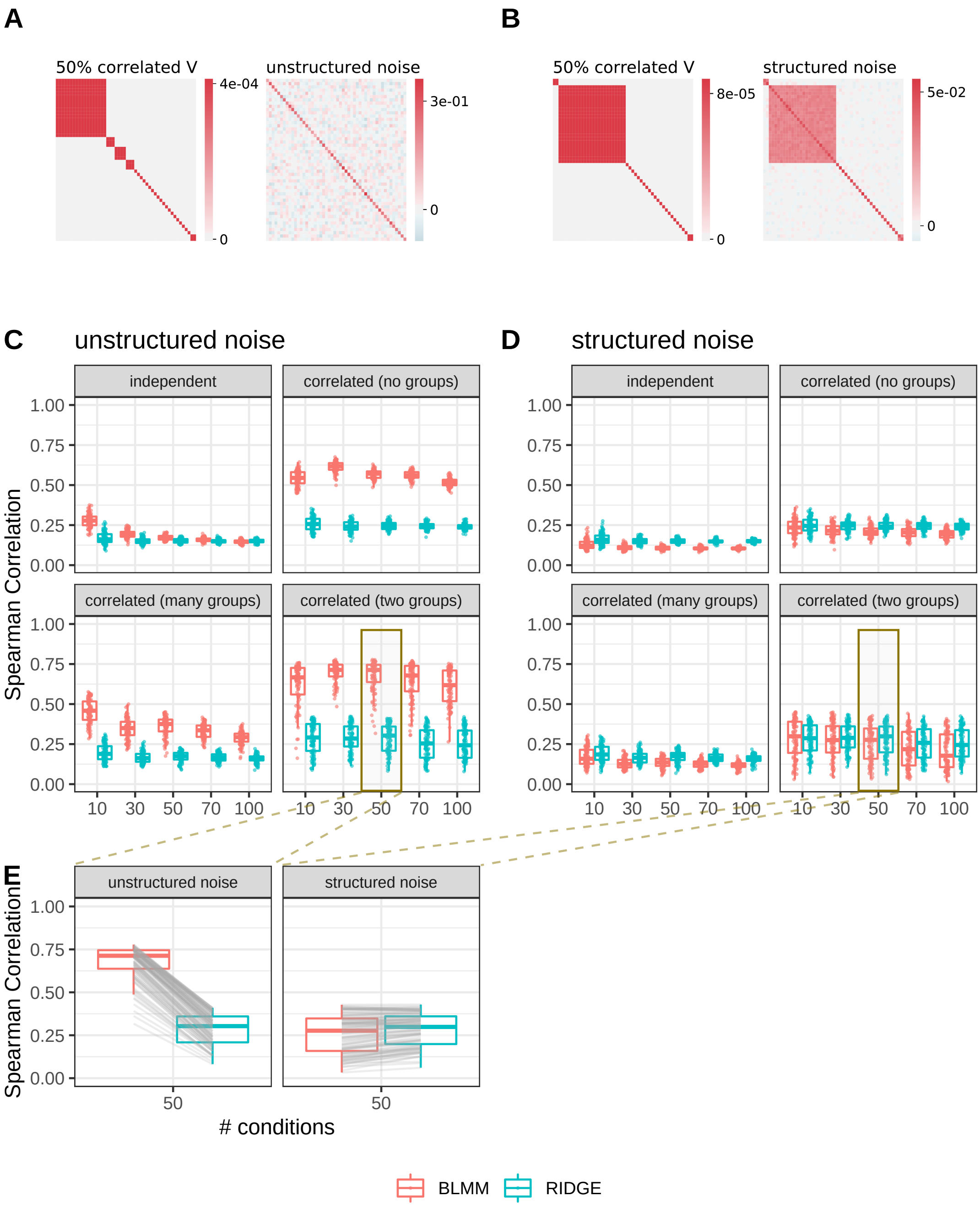

Figure S2: **Simulation Study with Spearman's rank correlation values** Results of simulation study analogously presented as in Fig. 2 with Spearman's rank correlation values.
