# Supplementary material for: Investigating the effect of dependence between conditions with Bayesian Linear Mixed Models for motif activity analysis
Source: PLoS One. 2020 May 1;15(5):e0231824. doi: 10.1371/journal.pone.0231824 (PMC7194367; doi:10.1371/journal.pone.0231824)
Supplement: S4 Fig — Data was generated over G = 978 informative genes, T = 623 motif scores, 100 repetitions, and with a covariance matrix “VC: highly correlated (two groups)”. ΣC is generated with increasing degree of structuredness (x-axis). Model performance of Bayesian Linear Mixed Model (BLMM, in red) and Ridge Regression (RIDGE, in blue) are shown on the bases of the Pearson correlation values between generated and predicted motif-condition-weights. (PDF) [file pone.0231824.s004.pdf]

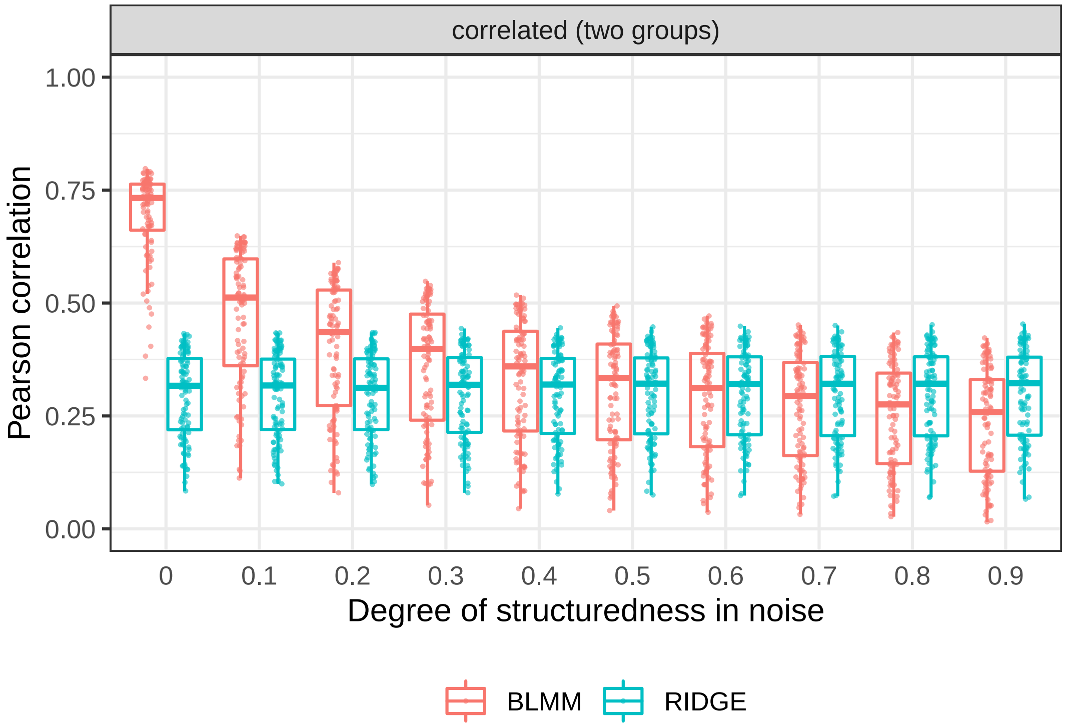

Figure S4: **Models' performance interchange superiority for data generated with increasing structuredness in noise** Data was generated over $G = 978$ informative genes, $T = 623$ motif scores, 100 repetitions, and with a covariance matrix "$\mathbf{V}_C$: highly correlated (two groups)". $\mathbf{\Sigma}_C$ is generated with increasing degree of structuredness (x-axis). Model performance of Bayesian Linear Mixed Model (BLMM, in red) and Ridge Regression (RIDGE, in blue) are shown on the bases of the Pearson correlation values between generated and predicted motif-condition-weights.
