# Supplementary material for: Investigating the effect of dependence between conditions with Bayesian Linear Mixed Models for motif activity analysis
Source: PLoS One. 2020 May 1;15(5):e0231824. doi: 10.1371/journal.pone.0231824 (PMC7194367; doi:10.1371/journal.pone.0231824)
Supplement: S5 Fig — We compare the model assumptions of Bayesian Linear Mixed Model (BLMM, depicted in red) and Ridge Regression (LIMIX, in blue) on datasets generated with C = 50 samples, and G = 5000 genes. On the x-axis we show data that is generated with unstructured noise (degree of structuredness = 0) and with structured noise with ρ = 0.7. On the y-axis, we depict the Pearson correlation values between generated and predicted motif-condition weights. In each panel, the data was generated with different assumptions on the degree of correlation between samples: (i) independence (VC = IC (upper left), (ii) unrestricted correlation (upper right), (iii) correlated with many sample groupgs (lower left), and (iv) highly correlated with two sample groups) (lower right). There is no difference in performance when increasing the dimensionality of genes. The Bayesian Linear Mixed Model has predictive power over Ridge Regression when the data is correlated, uniquely for unstructured noise. For structured noise (ρ = 0.7), there is no gain in performance, despite the bigger size of the dataset. (PDF) [file pone.0231824.s005.pdf]

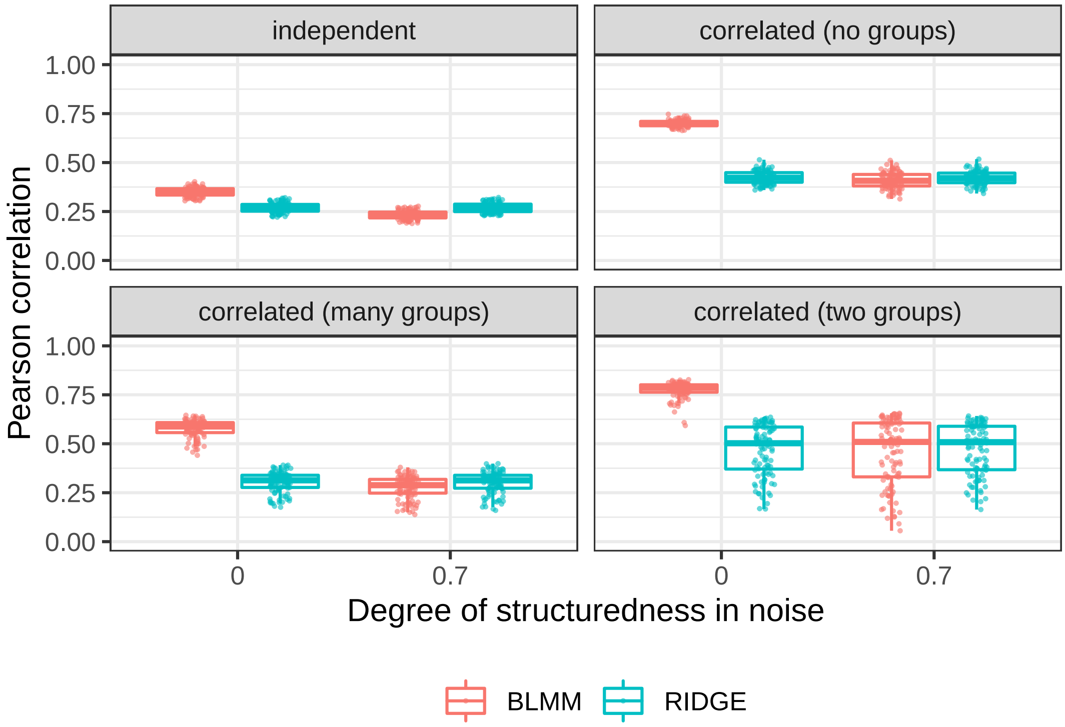

Figure S5: **Simulation study on $G = 5000$ genes** We compare the model assumptions of Bayesian Linear Mixed Model (BLMM, depicted in red) and Ridge Regression (LIMIX, in blue) on datasets generated with $C = 50$ samples, and $G = 5000$ genes. On the x-axis we show data that is generated with unstructured noise (degree of structuredness=0) and with structured noise with $\rho = 0.7$. On the y-axis, we depict the Pearson correlation values between generated and predicted motif-condition weights. In each panel, the data was generated with different assumptions on the degree of correlation between samples: (i) independence ($\mathbf{V}_C = \mathbf{I}_C$ (upper left), (ii) unrestricted correlation (upper right), (iii) correlated with many sample groupgs (lower left), and (iv) highly correlated with two sample groups) (lower right). There is no difference in performance when increasing the dimensionality of genes. The Bayesian Linear Mixed Model has predictive power over Ridge Regression when the data is correlated, uniquely for unstructured noise. For structured noise ($\rho = 0.7$), there is no gain in performance, despite the bigger size of the dataset.
