# Supplementary material for: Investigating the effect of dependence between conditions with Bayesian Linear Mixed Models for motif activity analysis
Source: PLoS One. 2020 May 1;15(5):e0231824. doi: 10.1371/journal.pone.0231824 (PMC7194367; doi:10.1371/journal.pone.0231824)
Supplement: S7 Fig — Estimated noise ΣC assuming independence between the conditions for the H3K27ac dataset. (PDF) [file pone.0231824.s007.pdf]

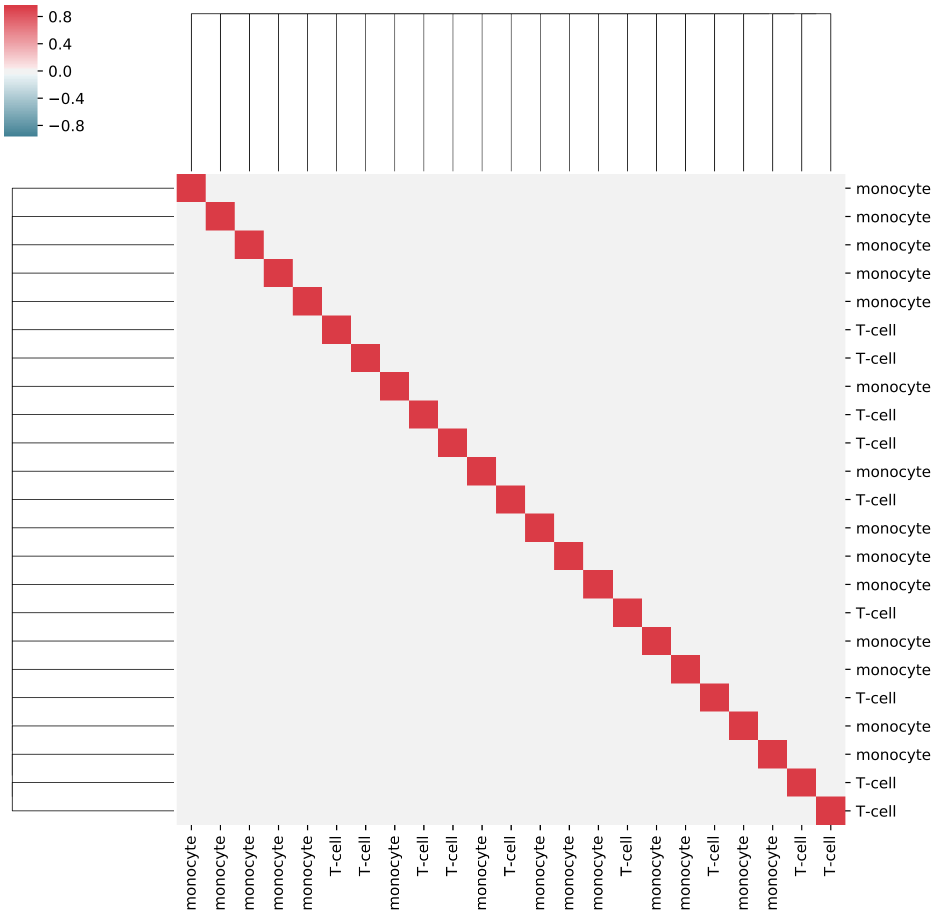

Figure S7: **H3K27ac: $\mathbf{\Sigma}_C$ for Ridge Regression** Estimated noise $\mathbf{\Sigma}_C$ assuming independence between the conditions for the H3K27ac dataset.
