# Supplementary material for: Investigating the effect of dependence between conditions with Bayesian Linear Mixed Models for motif activity analysis
Source: PLoS One. 2020 May 1;15(5):e0231824. doi: 10.1371/journal.pone.0231824 (PMC7194367; doi:10.1371/journal.pone.0231824)
Supplement: S8 Fig — Estimated correlation between conditions VC assuming dependence between the conditions for the H3K27ac dataset. (PDF) [file pone.0231824.s008.pdf]

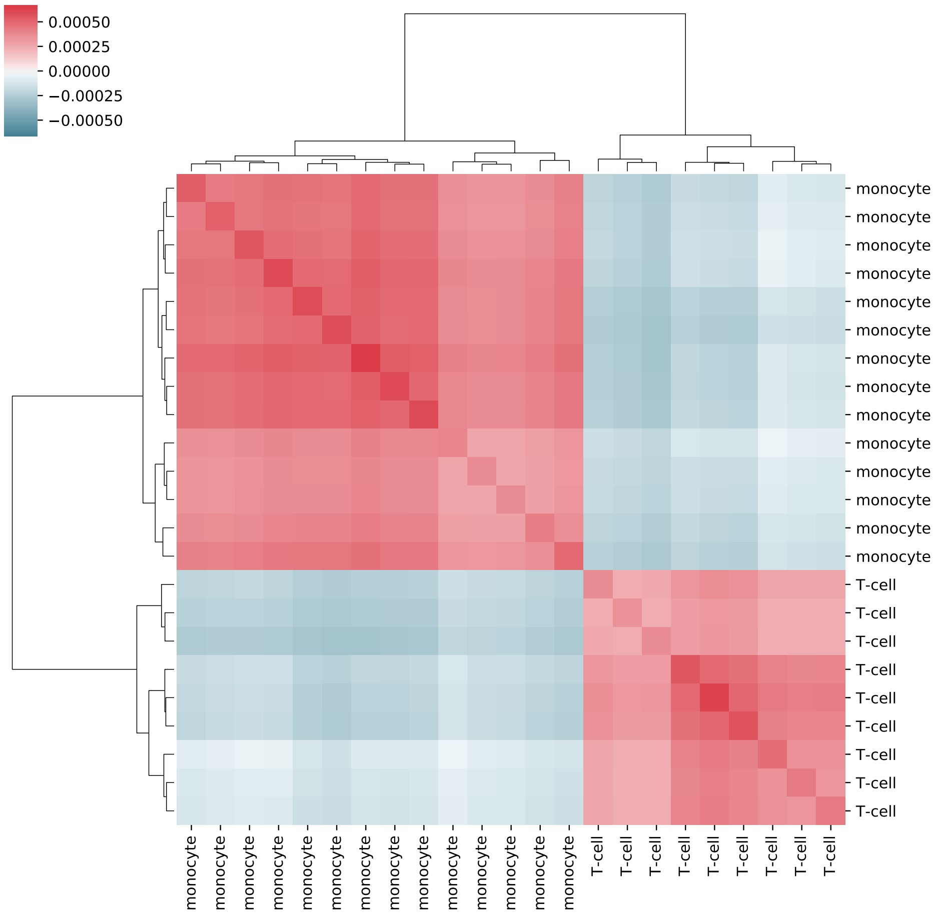

Figure S8: **H3K27ac: $\mathbf{V}_C$ for Bayesian Linear Mixed Model** Estimated correlation between conditions $\mathbf{V}_C$ assuming dependence between the conditions for the H3K27ac dataset.
