# Supplementary material for: Investigating the effect of dependence between conditions with Bayesian Linear Mixed Models for motif activity analysis
Source: PLoS One. 2020 May 1;15(5):e0231824. doi: 10.1371/journal.pone.0231824 (PMC7194367; doi:10.1371/journal.pone.0231824)
Supplement: S10 Fig — Weights for the motif-condition-weights of those 56 common motifs, that are outside of 2.5 times the inter-quantile range for all motifs over a tissue. The weights are computed assuming dependence (A) or independence (B) between the conditions. (PDF) [file pone.0231824.s010.pdf]

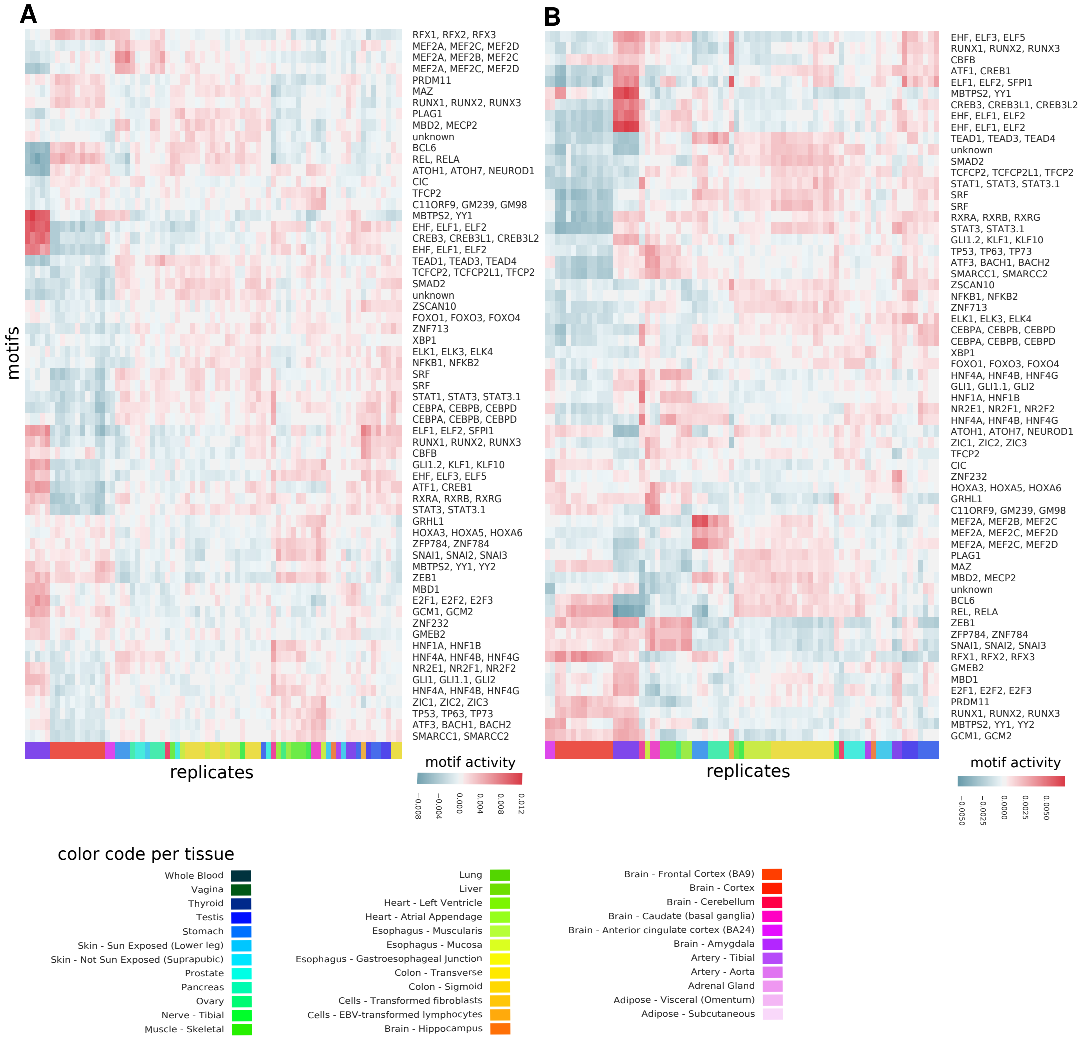

Figure S10: **GTEx: clustermaps of motif-condition-weight matrix** Weights for the motif-condition-weights of those 56 common motifs, that are outside of 2.5 times the interquantile range for all motifs over a tissue. The weights are computed assuming dependence (A) or independence (B) between the conditions.
