# Supplementary material for: Investigating the effect of dependence between conditions with Bayesian Linear Mixed Models for motif activity analysis
Source: PLoS One. 2020 May 1;15(5):e0231824. doi: 10.1371/journal.pone.0231824 (PMC7194367; doi:10.1371/journal.pone.0231824)
Supplement: S13 Fig — Estimated correlation between conditions VC assuming dependence between the conditions for the GTEx dataset. Note that only a subset of samples are labeled. (PDF) [file pone.0231824.s013.pdf]

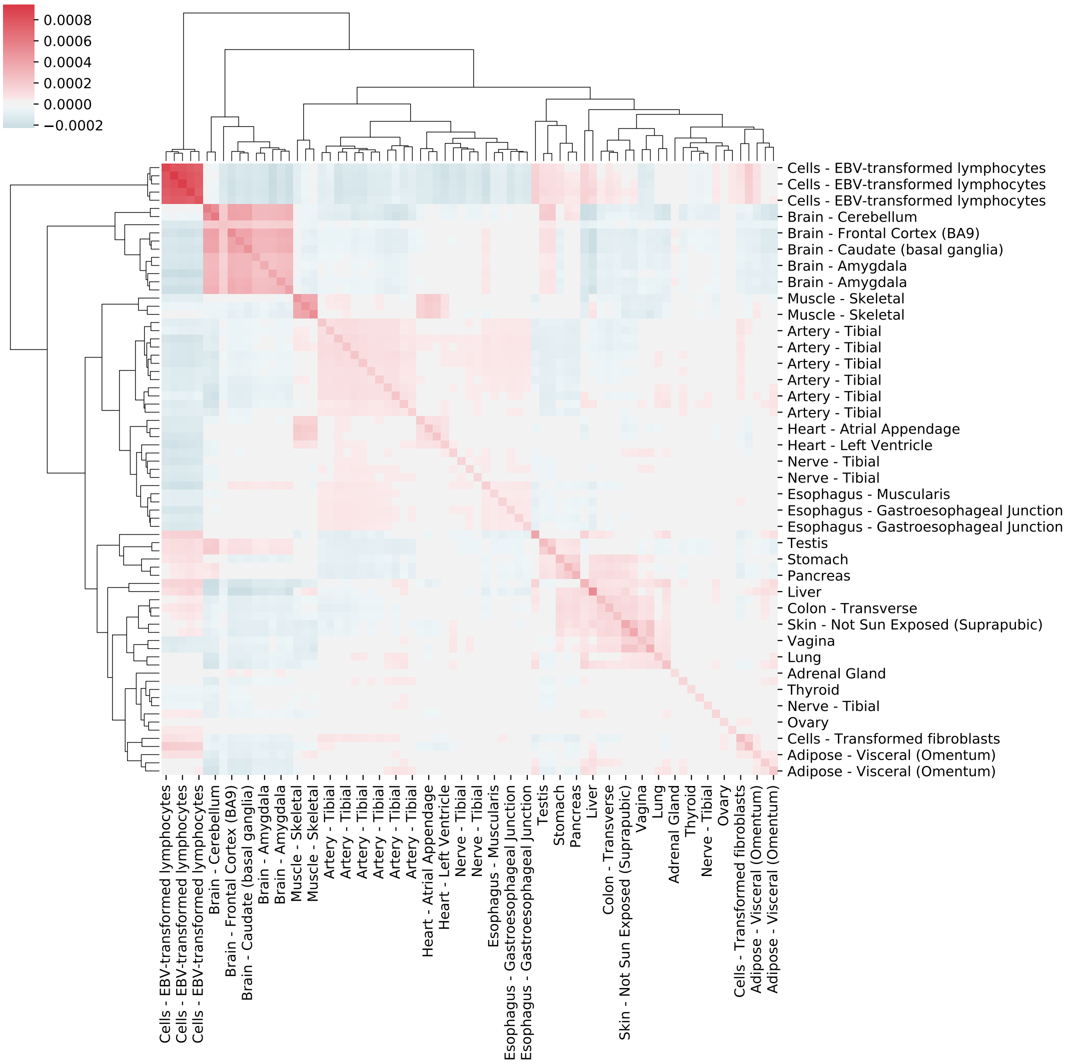


Figure S13: **GTEx: $\mathbf{V}_C$ for Bayesian Linear Mixed Model** Estimated correlation between conditions $\mathbf{V}_C$ assuming dependence between the conditions for the GTEx dataset. Note that only a subset of samples are labeled.
