# Supplementary material for: Investigating the effect of dependence between conditions with Bayesian Linear Mixed Models for motif activity analysis
Source: PLoS One. 2020 May 1;15(5):e0231824. doi: 10.1371/journal.pone.0231824 (PMC7194367; doi:10.1371/journal.pone.0231824)
Supplement: S14 Fig — Estimated noise ΣC assuming dependence between the conditions for the GTEx dataset. Note that only a subset of samples are labeled. (PDF) [file pone.0231824.s014.pdf]

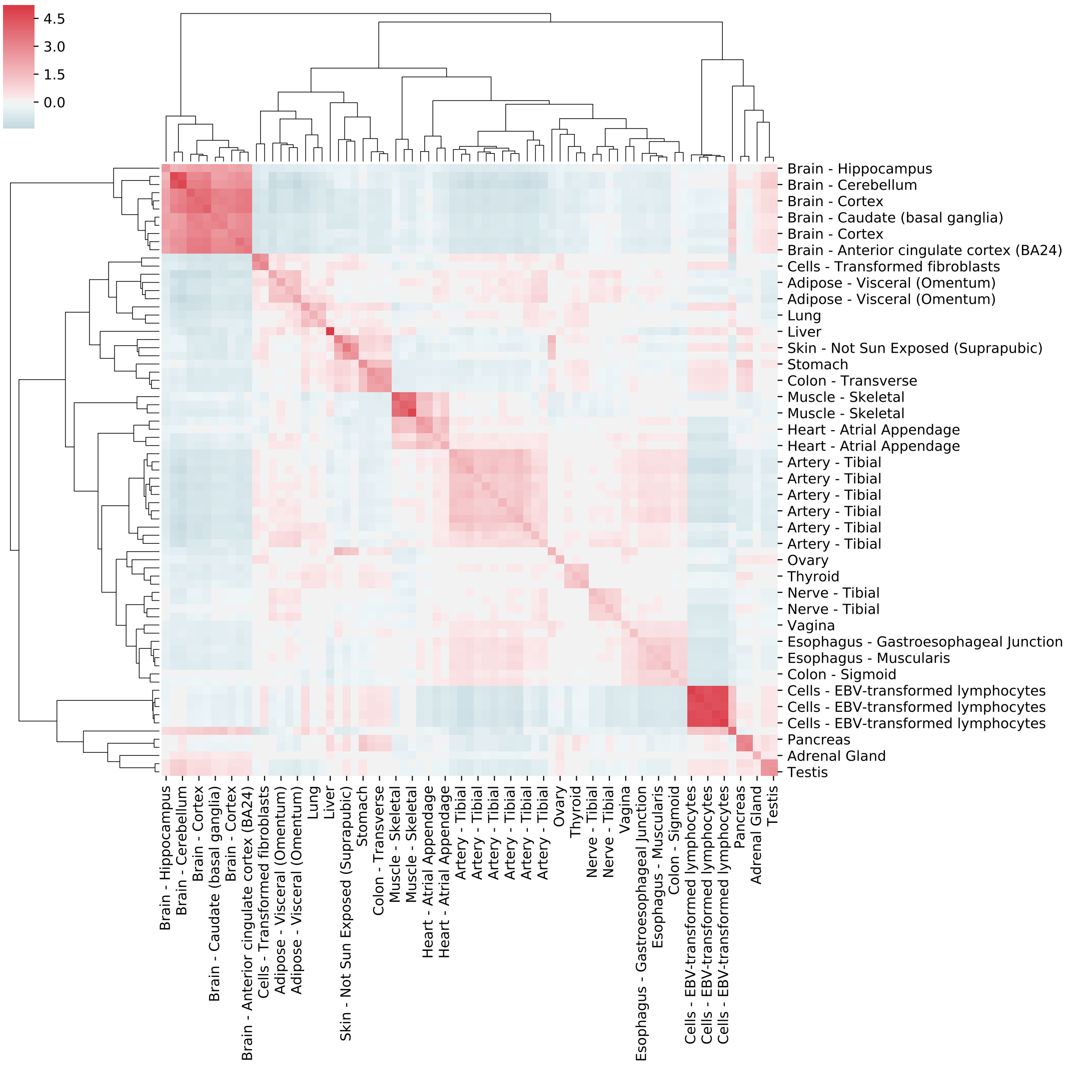

Figure S14: **GTEx: $\mathbf{\Sigma}_C$ for Bayesian Linear Mixed Model** Estimated noise $\mathbf{\Sigma}_C$ assuming dependence between the conditions for the GTEx dataset. Note that only a subset of samples are labeled.
