# Supplementary material for: Investigating the effect of dependence between conditions with Bayesian Linear Mixed Models for motif activity analysis
Source: PLoS One. 2020 May 1;15(5):e0231824. doi: 10.1371/journal.pone.0231824 (PMC7194367; doi:10.1371/journal.pone.0231824)
Supplement: S16 Fig — Motif values for the five lowest correlated motif scores over all tissues. (PDF) [file pone.0231824.s016.pdf]

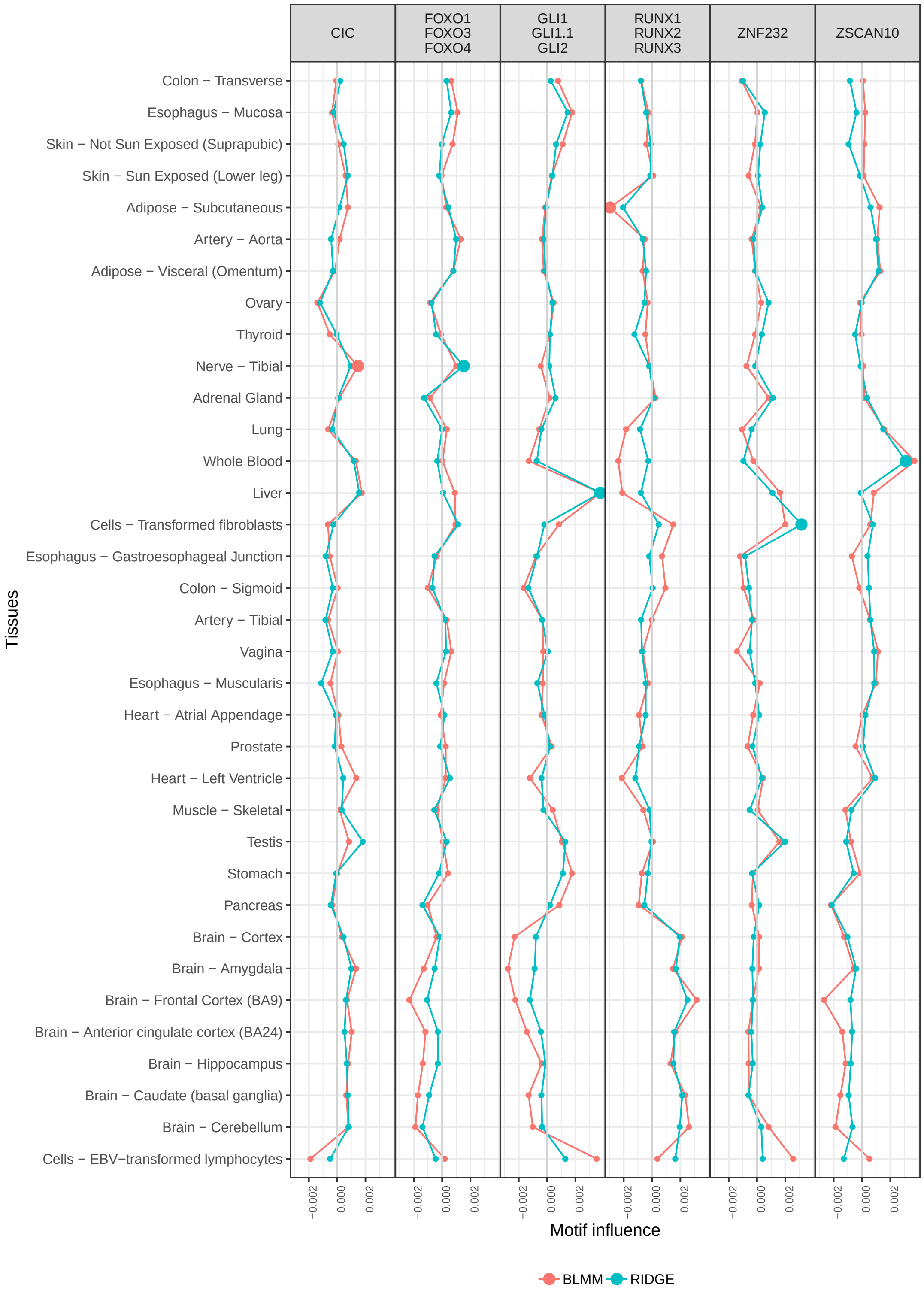

Figure S16: **GTEx: Least similar motif scores between methods** Motif values for the five lowest correlated motif scores over all tissues.
