# Supplementary material for: Investigating the effect of dependence between conditions with Bayesian Linear Mixed Models for motif activity analysis
Source: PLoS One. 2020 May 1;15(5):e0231824. doi: 10.1371/journal.pone.0231824 (PMC7194367; doi:10.1371/journal.pone.0231824)
Supplement: S20 Fig — Estimated noise ΣC assuming independence between the conditions for the Cacchiarelli dataset. (PDF) [file pone.0231824.s020.pdf]

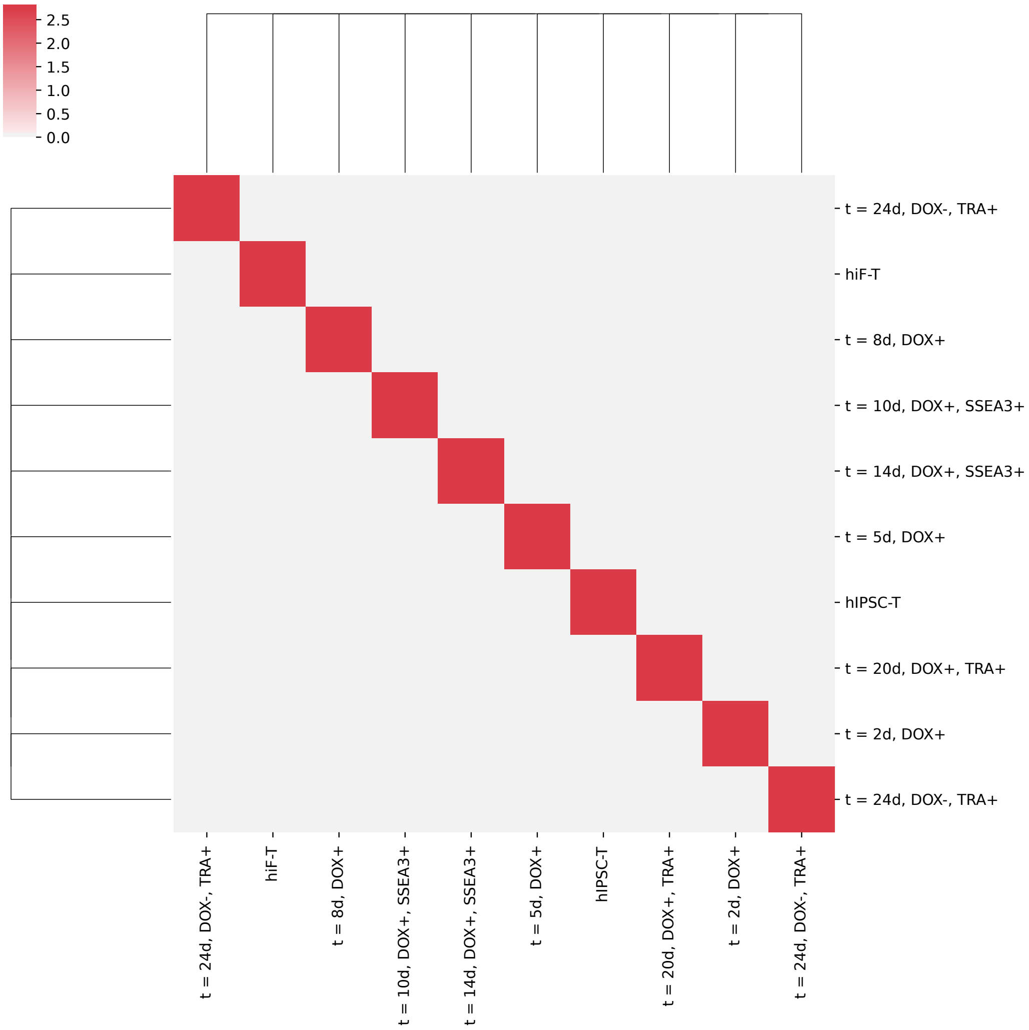

Figure S20: **Cacchiarelli: $\mathbf{\Sigma}_C$ for Ridge Regression** Estimated noise $\mathbf{\Sigma}_C$ assuming independence between the conditions for the Cacchiarelli dataset.
