# Supplementary material for: Investigating the effect of dependence between conditions with Bayesian Linear Mixed Models for motif activity analysis
Source: PLoS One. 2020 May 1;15(5):e0231824. doi: 10.1371/journal.pone.0231824 (PMC7194367; doi:10.1371/journal.pone.0231824)
Supplement: S23 Fig — Scatterplot of posterior motif weights ωT,C of Bayesian Linear Mixed Model vs. Ridge Regression, depicted per time series. (PDF) [file pone.0231824.s023.pdf]

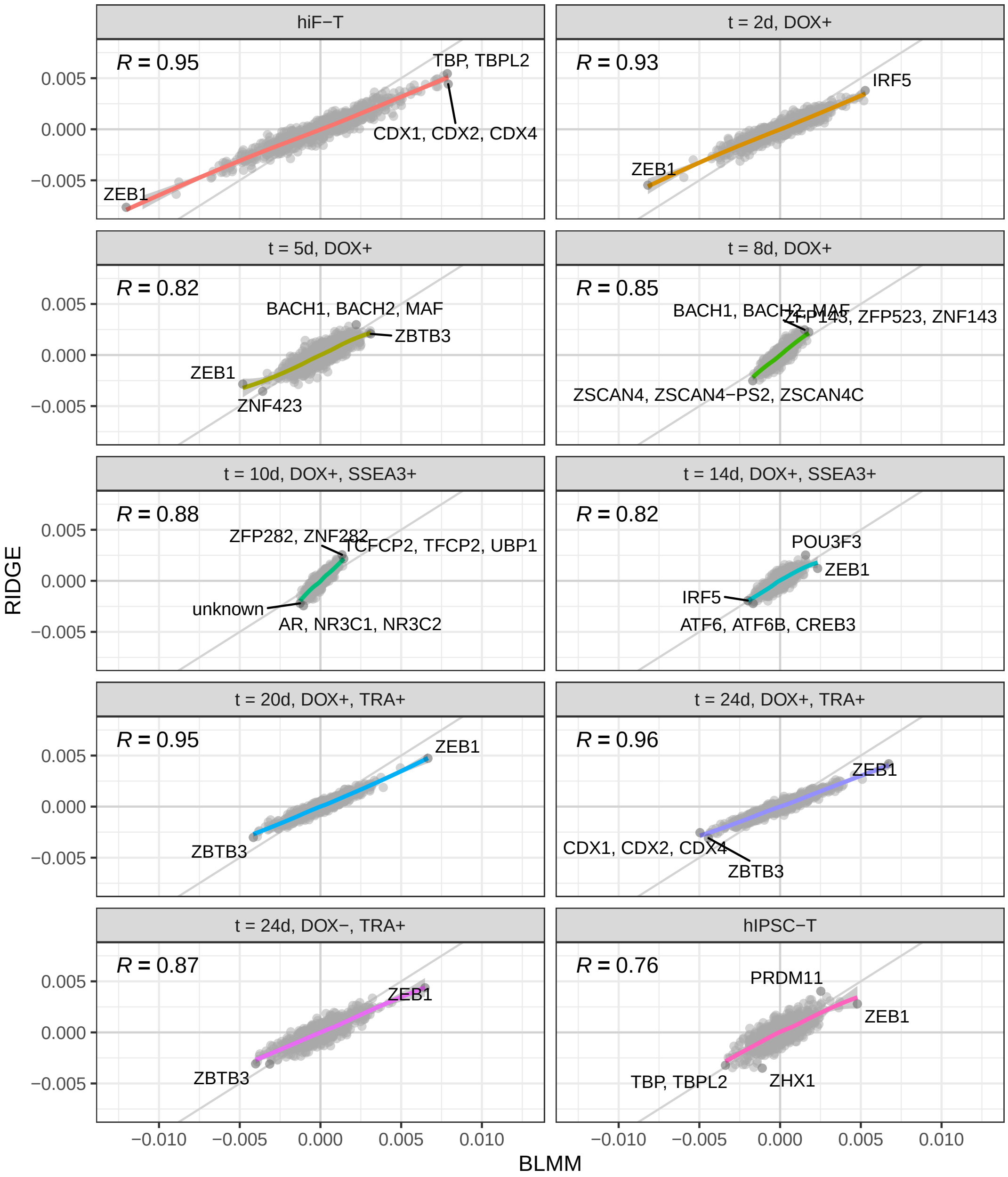

Figure S23: **Cacchiarelli: Scatterplot of estimated motif weights** $\omega_{T,C}$ Scatterplot of posterior motif weights $\omega_{T,C}$ of Bayesian Linear Mixed Model vs. Ridge Regression, depicted per time series.
