# Supplementary material for: Investigating the effect of dependence between conditions with Bayesian Linear Mixed Models for motif activity analysis
Source: PLoS One. 2020 May 1;15(5):e0231824. doi: 10.1371/journal.pone.0231824 (PMC7194367; doi:10.1371/journal.pone.0231824)
Supplement: S25 Fig — Estimated correlation between conditions VC assuming independence between the conditions for the Toufighi dataset. (PDF) [file pone.0231824.s025.pdf]

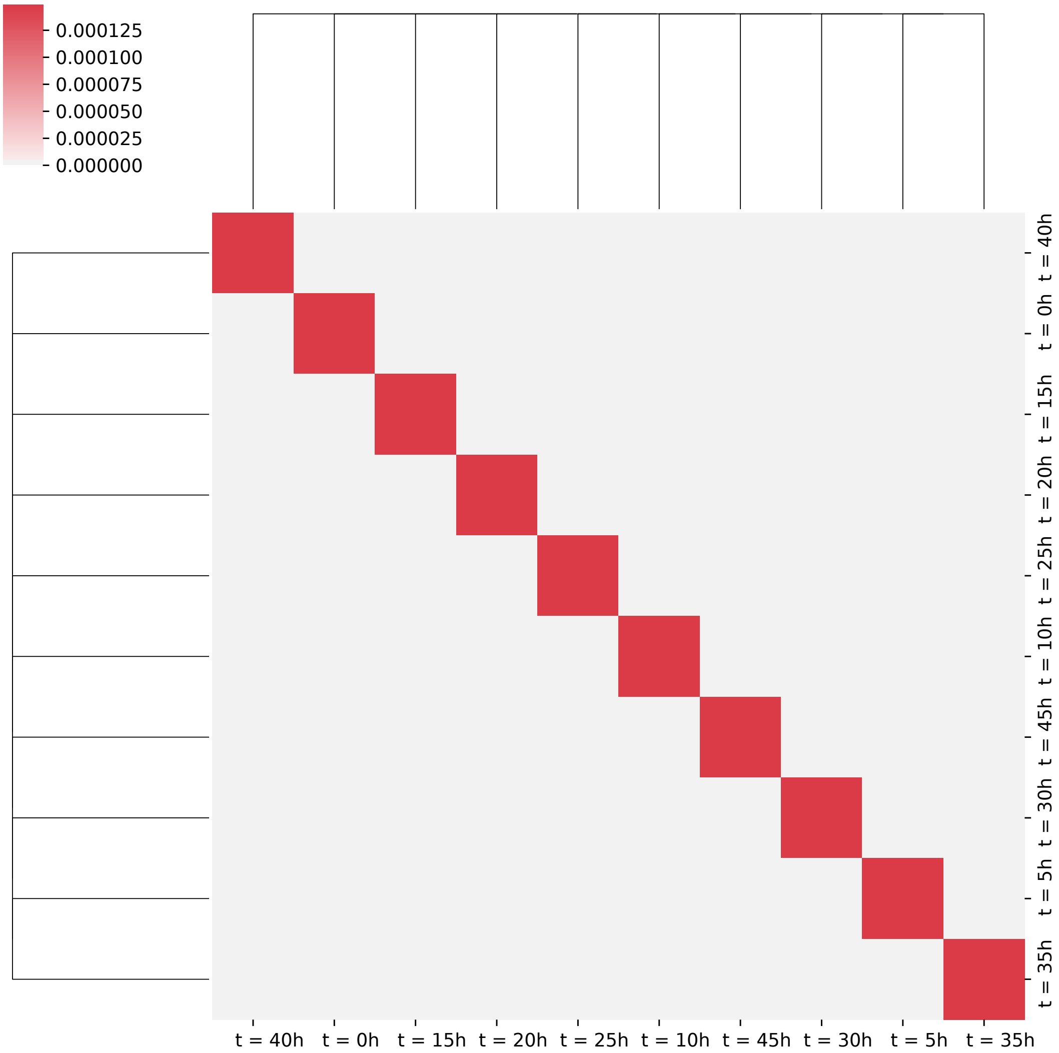

Figure S25: **Toufighi: $\mathbf{V}_C$ for Ridge Regression** Estimated correlation between conditions $\mathbf{V}_C$ assuming independence between the conditions for the Toufighi dataset.
