# Supplementary material for: Investigating the effect of dependence between conditions with Bayesian Linear Mixed Models for motif activity analysis
Source: PLoS One. 2020 May 1;15(5):e0231824. doi: 10.1371/journal.pone.0231824 (PMC7194367; doi:10.1371/journal.pone.0231824)
Supplement: S1 Appendix — We give a more detailed derivation of the model introduced in Eqs (1)–(4), providing more information about the Bayesian formulation. (PDF) [file pone.0231824.s030.pdf]

A Bayesian Linear Mixed Models

In this section, we explain in more detail the Bayesian take on the linear model

$$\bar{\mathbf{y}}_{g,c} = \sum_{t=1}^{T} \mathbf{m}_{t,g}\omega_{t,c} + \text{noise}, \tag{1}$$

where we model the normalized gene expression signal $\bar{\mathbf{y}}_{g,c} = \mathbf{y}_{g,c} - \bar{\mathbf{y}}_g - \bar{\mathbf{y}}_c$ as a linear combination of the motif scores $\mathbf{M}_{T,G}$, which are each, dependent on the sample c and the TF t, weighted with $\omega_{T,C}$. The term 'noise' represents all signal that cannot be explained by the model, i.e. the linear combination of the motif scores $\mathbf{M}_{T,G}$. This can be any technical noise, motif influence for which the linear assumption might be too simplistic, but also any other source that drives the gene expression $\mathbf{y}_{g,c}$ and is not modeled. The model was originally introduced by The Fantom Consortium and the Riken Omics Science Center (2009) and subsequently expanded by Balwierz *et al.* (2014).

The main idea behind a Bayesian approach is to include some prior knowledge into the data, called the prior. Here, we model $\omega_{t,c}$, the influence of motif $t \in \{1, \dots, T\}$ in condition $c \in \{1, \dots, C\}$, as a normal distributed prior with mean zero. Its marginal distributions are

$$\omega_C \sim \mathcal{N}\left(\mathbf{0}, \mathbf{V}_C\right), \tag{2}$$

where $\mathbf{V}_C$ is the covariance in motif activity over all conditions and

$$\omega_T \sim \mathcal{N}\left(\mathbf{0}, \sigma^{\mathbf{2}}\mathbf{I}_T\right), \tag{3}$$

with $\sigma^2\mathbf{I}_T$ being the covariance over all motifs. Hence, we assume independence between motifs. Assuming dependence between motifs with covariance $\mathbf{\Psi}$ can easily be implemented in the model. Making use of the vectorization representation of multivariate normal distribution, we can write the multivariate Normal distribution of $\omega_{T,C}$ as follows:

$$\text{vec}\left(\omega_{T,C}\right) \sim \mathcal{N}\left(\mathbf{0}, \sigma^{\mathbf{2}}\mathbf{V}_C \otimes \mathbf{I}_T\right). \tag{4}$$

Analogously, we can rewrite Eq. 1 in matrix-vector notation:

$$\text{vec}\left(\mathbf{Y}_{G,C}\right) \sim \mathcal{N}\left(\mathbf{M}_{T,G}^{\mathsf{T}}\omega_{T,C}, \delta\mathbf{\Sigma}_{C} \otimes \mathbf{I}_{G}\right), \tag{5}$$

with $\mathbf{\Sigma}_C$ being the covariance conditions and $\delta\mathbf{I}_G$ covariance over genes. In the following, we use Bayes' rule on marginal and conditional Gaussians (see **?**, Chapter 2.3, p. 93). We first list the general formulas here, which we copy from **?**: The marginal distribution of $\mathbf{x}$ and conditional distribution of $\mathbf{y}$ given $\mathbf{x}$ are given as follows:

$$p(\mathbf{x}) = \mathcal{N}\left(\mathbf{x}|\mu, \mathbf{\Lambda}^{-1}\right) \tag{6}$$

$$p(\mathbf{y}|\mathbf{x}) = \mathcal{N}\left(\mathbf{y}|\mathbf{A}\mathbf{x} + \mathbf{b}, \mathbf{L}^{-1}\right). \tag{7}$$

Translating that notation into our own notation, we get the following equivalences:

$$\mathbf{x} \equiv \text{vec}\left(\omega_{T,C}\right) \qquad \mu \equiv \mathbf{0} \qquad \mathbf{\Lambda}^{-1} \equiv \sigma^{\mathbf{2}}\mathbf{V}_C \otimes \mathbf{I}_T \tag{8}$$

$$\mathbf{y} \equiv \text{vec}\left(\mathbf{Y}_{G,C}\right) \qquad \mathbf{A} \equiv \mathbf{M}_{T,G}^{\mathsf{T}} \qquad \mathbf{b} \equiv \mathbf{0} \qquad \mathbf{L}^{-1} \equiv \delta\mathbf{\Sigma}_C \otimes \mathbf{I}_G. \tag{9}$$

In our own notation, and following Eq. 7, the distribution of $\mathbf{Y}_{G,C}$ given $\omega_{T,C}$ can be rewritten as:

$$\text{vec}\left(\mathbf{Y}_{G,C}|\omega_{T,C}\right) \sim \mathcal{N}\left(\mathbf{0}, \sigma^{\mathbf{2}}\mathbf{V}_C \otimes \mathbf{\Pi}_G + \delta\mathbf{\Sigma}_C \otimes \mathbf{I}_G\right), \tag{10}$$

where $\mathbf{\Pi}_G = \mathbf{M}_{T,G}^{\mathsf{T}}\mathbf{M}_{T,G}$.

Going back to the general formula from **?**, the marginal distribution of $\mathbf{y}$ and conditional distribution of $\mathbf{x}$ given $\mathbf{y}$ are computed according to the following:

$$p(\mathbf{y}) = \mathcal{N}\left(\mathbf{y}|\mathbf{A}\mu + \mathbf{b}, \mathbf{L}^{-1} + \mathbf{A}\mathbf{\Lambda}^{-1}\mathbf{A}^T\right) \tag{11}$$

$$p(\mathbf{x}|\mathbf{y}) = \mathcal{N}\left(\mathbf{x}|\mathbf{\Sigma}\left\{\mathbf{A}^T\mathbf{L}\left(\mathbf{y} - \mathbf{b}\right) + \mathbf{\Lambda}\mu\right\}, \mathbf{\Sigma}\right), \tag{12}$$

$$\text{with } \mathbf{\Sigma} = \left(\mathbf{\Lambda} + \mathbf{A}^T\mathbf{\Lambda}\mathbf{A}\right)^{-1}. \tag{13}$$

Hence, based on Eq. 11 and Eq. 12, the posterior distribution of $\omega_{T,C}$ given $\mathbf{Y}_{G,C}$ is then

$$\text{vec}\left(\omega_{T,C}|\mathbf{Y}_{G,C}\right) \sim \mathcal{N}\left(\left(\mathbf{V}_C \otimes \mathbf{M}_{T,G}^{\mathsf{T}}\mathbf{I}_T\right)\mathbf{\Lambda_{CG\otimes CG}}^{-1}\text{vec}\left(\mathbf{Y}_{G,C}\right), \mathbf{\Lambda_{CG\otimes CG}}\right) \tag{14}$$

with

$$\mathbf{\Lambda_{CG\otimes CG}} = \sigma^{\mathbf{2}}\mathbf{V}_C \otimes \mathbf{\Pi}_G + \delta\mathbf{\Sigma}_C \otimes \mathbf{I}_G. \tag{15}$$

For the reformulation, we used the *Woodbury matrix identity* (Woodbury, 1950). For the computation of the posterior values of $\omega_{T,C}$, with notation $\hat{\omega}_{T,C}$, it suffices to compute the mean of Eq. 14:

$$\text{vec}\left(\hat{\omega}_{T,C}|\mathbf{Y}_{G,C}\right) = \left(\mathbf{V}_C \otimes \mathbf{M}_{T,G}^{\mathsf{T}}\mathbf{I}_T\right)\left[\sigma^{\mathbf{2}}\mathbf{V}_C \otimes \mathbf{\Pi}_G + \delta\mathbf{\Sigma}_C \otimes \mathbf{I}_G\right]^{-1}\text{vec}\left(\mathbf{Y}_{G,C}\right) \tag{16}$$

As the covariance matrix is assumed to be the sum of Kronecker products, the runtime complexity is reduced to $O\left(G^3 + C^3\right)$ in a $O\left(G^2 + C^2\right)$ space instead of $O\left(G^3C^3\right)$ runtime and a memory requirement of $O\left(G^2C^2\right)$ (Lippert *et al.*, 2014).
