# Supplementary material for: Investigating the effect of dependence between conditions with Bayesian Linear Mixed Models for motif activity analysis
Source: PLoS One. 2020 May 1;15(5):e0231824. doi: 10.1371/journal.pone.0231824 (PMC7194367; doi:10.1371/journal.pone.0231824)
Supplement: S2 Appendix — Extensive details about the simulation of data. (PDF) [file pone.0231824.s031.pdf]

B Simulating data

For the simulation study we generate data based on the model introduced in Eq. 1-Eq. 4, given a covariance matrix $\mathbf{V}_C$. The prior weight $\tilde{\omega}_{T,C}$ and $\mathbf{Y}_{G,C}$ are generated according to Eq. 2 and Eq. 1. The expression data $\mathbf{Y}_{G,C}$ from Eq. 1 is then used to estimate $\mathbf{V}_C$ and $\mathbf{\Sigma}_C$. Based on these computations, the posterior motif influence $\hat{\omega}_{T,C}$ is then computed and compared to the simulated motif influence $\tilde{\omega}_{T,C}$ with a Pearson correlation (Pearson, 1895) over all conditions. As gene set we use the 978 landmark genes from the LINCS project (Koleti *et al.*, 2018). In a secondary simulation we increase the size of the gene set to 5000 genes, which originate from an analysis of the most variational genes across all samples from the GTEx project (Genotype Tissue Expression, `https://gtexportal.org/home/`). We generate data for $C = \{10, 30, 50, 70, 100, 120\}$ conditions.

Covariance types of $\mathbf{V}_C$

For the generation of simulated motif influence $\tilde{\omega}_{T,C}$ (Eq. 2), a covariance matrix $\sigma^2\mathbf{V}_C \otimes \mathbf{I}_T$ needs to be given. As the covariance along TFs is modeled to be independent, one can generate $\tilde{\omega}_{t,C}$ randomly T times with $\tilde{\omega}_{t,C} \sim \mathcal{N}\left(0, \sigma^2\mathbf{V}_C\right)$. In the following, four covariance types are explained that we generate as $\mathbf{V}_C$ to serve as covariance for the generated $\omega_{T,C}$ in Eq. 2 and hence expression data $\mathbf{Y}_{G,C}$ in Eq. 3. All covariance matrices are then normalized by their trace. Below, we explain how σ^2 is generated.

Independence

For independent conditions, we simply set $\mathbf{V}_C$ to an identity matrix: $\mathbf{V}_C = \sigma^2\mathbf{I}_C$. We refer to these as independent, but in reality they are even isotropic, as the covariance between samples is assumed to be identical.

Unrestricted correlation

A random (uniform) $C \times C$ matrix $\mathbf{R}_C$ is generated and added with an identity matrix. This is then multiplied with itself: $\mathbf{V}_C = \sigma^2\left(\mathbf{I}_C + \mathbf{R}_C\right)\left(\mathbf{I}_C + \mathbf{R}_C\right)^{\intercal}$

Correlation - high correlation and 50% correlation

We assume correlation between samples to allow for replicates and similarities between cell-lines. Hence, we would expect that those samples cluster in blocks when applying a clustering algorithm to the data (see Fig. S8 or Fig. S13 as examples). Therefore, we generate these block matrices with k blocks of ones. Samples within are block are then completely correlated and completely independent from samples outside that block. The size of the blocks is minimally one and randomly determined. The sum of all block sizes is C. These blocks are lined up along the diagonal. The non-block elements are set to -0.01. The procedure is written in pseudo-code in Supplement B. For an example of a $k = \frac{1}{2}C$ covariance matrix, see Fig. 2A and B, left panel. For the analysis, we generate highly correlated covariance matrices with two blocks, i.e. $k = 2$, and 50% correlated covariance matrices, they are hence of medium rank, with $k = \frac{1}{2}C$ correlated sample groups.

Noise $\mathbf{\Sigma}_C$ - unstructured and structured

To generate the gene expression data $\mathbf{Y}_{G,C}|\omega_{T,C}$, we compute the signal as the product of the motif scores $\mathbf{M}_{T,G}$ (explained hereafter) and $\omega_{T,C}$ (explained previously). Due to the randomness in the signal that is not explained by motifs, we add some random noise which is drawn from a normal distribution with covariance $\delta\mathbf{\Sigma}_C \otimes \mathbf{I}_G$. We generate $\mathbf{\Sigma}_C$ in two different ways: (i) assuming no particular structure, $\mathbf{\Sigma}_{C,\text{random}}$, which is a matrix filled with values drawn from a standard normal distribution, multiplied with itself, or (ii) adding structure

that is similar to $\mathbf{V}_C$: $\mathbf{\Sigma}_{C,\mathbf{V}_C,\rho} = \mathbf{\Sigma}_{C,\text{random}} + \eta_\rho \mathbf{V}_C$. The noise matrices are then normalized by their trace.

Unstructured noise $\mathbf{\Sigma}_C$

In a first step, we randomly generate a covariance matrix, analogously to the random covariance matrix $\mathbf{V}_C$: by taking the product of a randomly drawn matrix, we assure its symmetry and positive definiteness. A visualization is given in Fig. 2A, right panel.

Structured noise $\mathbf{\Sigma}_C$

To the unstructured noise matrix $\mathbf{\Sigma}_C$, explained in the previous paragraph, we add a structure that is similar to $\mathbf{V}_C$:

$$\mathbf{\Sigma}_{C,\mathbf{V}_C,\rho} = \eta_\rho \mathbf{V}_C + \mathbf{\Sigma}_{C,\text{random}}, \tag{17}$$

where η_ρ depends on $\rho \in [0, 1]$, the degree of structure in the noise. If $\rho = 0$, the noise is unstructured, and a fraction ρ of one yields the sum of $\mathbf{V}_C$ and $\mathbf{\Sigma}_C$, normalized by their traces, respectively:

$$\eta_\rho = \begin{cases} 0 & \text{if } \rho = 0 \\ \frac{\rho}{1-\rho} \frac{\text{tr}(\mathbf{\Sigma}_C)}{\text{tr}(\mathbf{V}_C)} & \text{for } \rho \in (0, 1) \end{cases} \tag{18}$$

$$\mathbf{\Sigma}_{C,\mathbf{V}_C,\rho} = \eta_\rho \mathbf{V}_C + \mathbf{\Sigma}_{C,\text{random}} \tag{19}$$

We therefore control the degree of structure in the noise. A visualization of such a structured noise matrix is given in Fig. 2B, right panel. As any model is just a simplification of reality, we cannot explain the entire signal expressed in the expression data $\mathbf{Y}_{G,C}$. This is especially the case as we model the signal uniquely as a linear product of artificially computed motif scores. Hence, there will always be signal in the data that cannot be explained by motifs. We therefore add to the random environmental or technical noise signal that explains the relationship between conditions.

Signal-to-noise ratio

From previous research (Balwierz *et al.*, 2014), it has been shown that roughly $10 - 20\%$ of the signal of gene expression can be explained by motif influence in the promoter region. We therefore generate the data in such a way, that 20% of the signal in expression data $\mathbf{Y}_{G,C}$ is due to motifs, and the rest unexplainable noise. We achieve this by adjusting the parameter $\sigma^{\mathbf{2}}$ and δ. The latter is fixed by the rough percentage wished to be expressed by the noise, $1 - \beta$, which we set to $\beta = 0.2$. For scaling reasons, it is divided with the 2-norm of $\mathbf{\Sigma}_C$:

$$\delta = \frac{(1-\beta)}{\|\mathbf{\Sigma}_C\|_2}, \tag{20}$$

with $\|\cdot\|_2 = \sigma_{\max}(\cdot)$ as the matrix 2-norm which is equivalent to $\sigma_{\max}(\cdot)$, the maximal singular value. $\sigma^{\mathbf{2}}$ is determined by bisection, such that it explains 0.2 of the variance coefficient (Eq. 1). As starting value, we set:

$$\sigma^{\mathbf{2}} = \delta \frac{\beta}{1-\beta} \frac{C \text{tr}(\mathbf{\Sigma}_C)}{\text{tr}(\mathbf{V}_C)\, \text{tr}(\mathbf{\Pi}_G)}. \tag{21}$$

Pseudocode for the generation of lower rank block matrices

Algorithm 1 Generation of correlated covariance matrices

```
function generateBlocksizes( C, k )          ▷ generate k blocksizes that sum up to C
    num_blocks ← k
    blocksizes ← initialize vector of length num_blocks
    for block_i in (num_blocks − 1) do
        lenght_blocks ← sum( blocksizes )
        leftover-space ← C − length_blocks + 1
        blocksize_i ← random integer between 1 and leftover-space
        blocksizes[i] ← blocksize_i
    blocksizes[num_blocks] ← C − block-lengths + 1
    return blocksizes
procedure Block matrix of dimension C with k blocks of size blocksizes
    matrix ← initialize matrix of dimension C
    block_i_start ← 1
    block_i_end ← 0
    blocksizes ← generateBlocksizes(C, k)
    for blocksize_i in blocksizes do
        block_i_end ← block_i_end + blocksize_i
        matrix[block_i_start : block_i_end, block_i_start : block_i_end] ←
            matrix of ones of size blocksize_i        ▷ place matrix of ones onto diagonal
        block_i_start ← block_i_end
    for all elements in matrix that are zero do
        fill with 1e−2                                ▷ fill all off-diagonal elements
```

References

Balwierz, P. J. *et al.* (2014). ISMARA: automated modeling of genomic signals as a democracy of regulatory motifs. *Genome Res.*, **24**(5), 869–884.

Koleti, A. *et al.* (2018). Data Portal for the Library of Integrated Network-based Cellular Signatures (LINCS) program: integrated access to diverse large-scale cellular perturbation response data. *Nucleic Acids Research*, **46**(D1), D558–D566.

Lippert, C. *et al.* (2014). Supplemental Information Multivariate analysis of heritable traits. *bioRxiv*.

Pearson, K. (1895). VII. Note on regression and inheritance in the case of two parents. *Proceedings of the Royal Society of London*, **58**(347-352), 240–242.

The Fantom Consortium and the Riken Omics Science Center (2009). The transcriptional network that controls growth arrest and differentiation in a human myeloid leukemia cell line. *Nature Genetics*, **41**(5), 553–562.

Woodbury, M. A. (1950). Inverting modified matrices. *Memorandum report*, **42**(106), 336.
