# Supplementary material for: Investigating the effect of dependence between conditions with Bayesian Linear Mixed Models for motif activity analysis
Source: PLoS One. 2020 May 1;15(5):e0231824. doi: 10.1371/journal.pone.0231824 (PMC7194367; doi:10.1371/journal.pone.0231824)
Supplement: S24 Fig — Motif weights for the 50 most variable weights across conditions assuming dependence (A) or independence (B) between the conditions. Common motifs of Bayesian Linear Mixed Model (A) and Ridge Regression (B) are depicted in blue, others are depicted in yellow. (PDF) [file pone.0231824.s024.pdf]

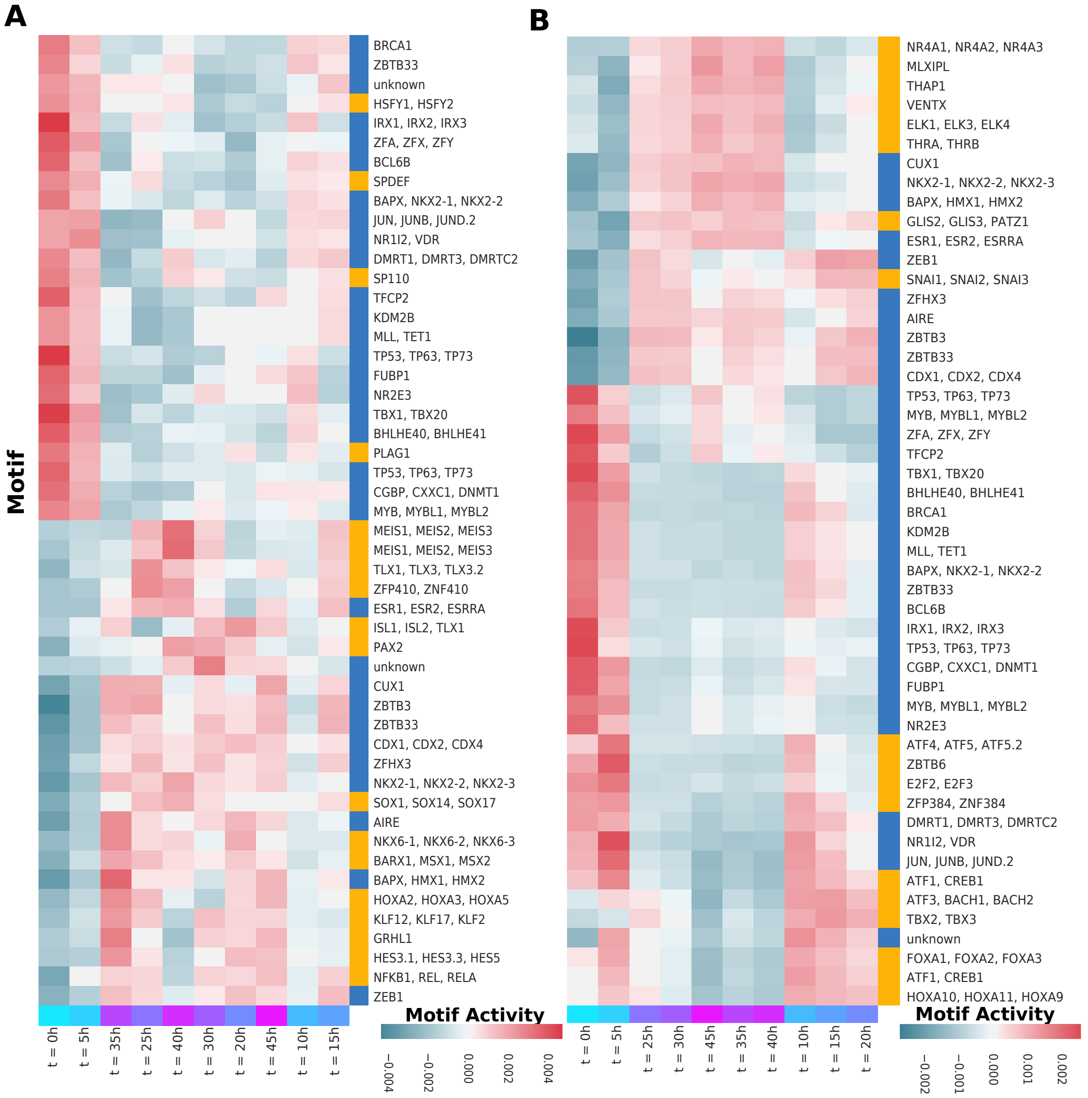

Figure S24: **Toufighi: clustermaps of motif-condition-weight matrix** Motif weights for the 50 most variable weights across conditions assuming dependence (A) or independence (B) between the conditions. Common motifs of Bayesian Linear Mixed Model (A) and Ridge Regression (B) are depicted in blue, others are depicted in yellow.
