# Supplementary material for: Investigating the effect of dependence between conditions with Bayesian Linear Mixed Models for motif activity analysis
Source: PLoS One. 2020 May 1;15(5):e0231824. doi: 10.1371/journal.pone.0231824 (PMC7194367; doi:10.1371/journal.pone.0231824)
Supplement: Attachment — Submitted filename: PLOSOne_Letter_to_reviewers.pdf [file pone.0231824.s032.pdf]

Tuebingen, February 29th , 2020

Dear Editor,
Dear Reviewers,

We would like to submit our revised manuscript entitled **Investigating the Effect of Dependence between Conditions in Bayesian Linear Mixed Models for Motif Activity Analysis** by Simone Lederer, Simon J van Heeringen, Tom Heskes and Cornelis A Albers.
We thank the reviewers for their comments which we believed helped to make the paper more understandable, highlight the results better and helps the reader to navigate better through the paper. We have carefully taken all comments into consideration and would like to address them in this letter and explain to you in detail how we proceeded.
We submit two versions of the revised article. The first is the revised version of the article. In the second we highlight all changes made in the text. Please note that we did not highlight changes in tables or figures. We hope that this second version helps the reviewers better to identify the changes we made based on their remarks.

Reviewer 1
Major concerns

1. *The title of the manuscript seems to indicate they are introducing a novel useful model for motif analysis. However the novelty is limited and the model is not useful on real data as they state in the abstract.*
   We agree with the reviewer, that the original title "**Bayesian Linear Mixed Models for Motif Activity Analysis**" might have been misleading. We changed the title to "**Investigating the Effect of Dependence between Conditions in Bayesian Linear Mixed Models for Motif Activity Analysis**".

2. *The number of real datasets tested on is only 2 which makes any implication that modeling correlation is not useful suspect.*
   We introduced two more datasets (details below in the following paragraph) and show that the results from the simulation study do indeed hold true on real datasets. We would also like to point out that we have a mathematical explanation for why modeling correlation doesn't work well in practice.

3. *The authors could test their method on time series data. There are several models that successfully incorporate temporal dependence and it would be a good test of their methodology to check if they find no advantage in modeling the correlation there as well.*
   We thank the reviewer for the suggestion. We additionally analyzed two datasets on time series data. We chose two datasets which measure gene expression changes of *in vitro* cultured cells of two well-characterized systems, RNA-seq data of transdifferentiation from fibroblast to pluripotent cells [Cacchiarelli et al, 2015] and differentiation of keratinocytes measured using micro-array data [Toufighi et al., 2015].

4. *The story needs to be made more clear. The manuscript should either be geared towards the introduction of a new model and showing its advantages or testing whether modeling the dependence between samples helps in which case it would be useful to test more models with and without the dependence modeling as well as test on more datasets. Testing on time series data may be a good control.*
   In our proposed article we focus on the extension of a widely used model, the Ridge Regression, to allow for dependencies between conditions. We think it is important to introduce the model extensively for the reader to be able follow up and understand the

results, that are presented. We do extensively test the model on four datasets in total and show that the results of the simulation study are consistent with real world data. We additionally give an explanation to why the Bayesian Linear Mixed Model does not perform significantly better. We consider this detail to be of importance for other researchers to consider in further modeling approaches.

Reviewer 2

Major comments:

1. *It would be helpful if the authors explained to the reader what is novel and what is confirmatory.*

   We developed the mathematical framework to allow for correlation/covariance between samples and applied it to predicting motif activity. Predicting motif activity has hitherto only been done with Ridge Regression. We rewrote parts of the discussion and conclusion to better reflect this.

Specific comments

2. Results:
   a. *Authors didn't elaborate on how they chose parameters for models.*

      We stated the parameter settings chosen in section Implementation and model testing. For a better navigation, we separated this section into three different sections, Code availability, Model fitting, and Visualization. In Model fitting, we give, to the best of our knowledge, all important details about the initial parameter settings and their computation.

   b. *How robust is results for both models?*

      We used 100 repetitions in the simulation study, where the ground truth is known, and apply cross-validation on both datasets where the ground truth is not known, to show the robustness of both models.

   c. *Can authors randomize motif hits on real datasets and compare performance for both methods?*

      We now show results for randomized motif scores in every analysis. As expected, randomized motifs have no predictive performance, as measured in the test data set during cross-validation.

   d. *Can authors report spearman correlation as well as MSE performance?*

      We included the Spearman rank correlation and MSE values in the Supplement, Fig. S8, Fig, S9

   e. *Result section would be benefit from the addition of another dataset where the ground truth is know and compare methods*

      see comments 2-4 of reviewer 1. It is not possible to find datasets with known ground-truth, but we extended the analysis by two more datasets that incorporate time series. The lack of ground truth data for gene regulatory network analysis is a problem in the field. While we wholeheartedly agree with the reviewer that this would be highly beneficial, it falls outside the scope of this work to generate such a data set.

3. Figures:
   a. *Please give a description about the framework in Figure 1 legend.*

      We thank the reviewer for the comment and included a description to the project overview in Figure 1

We hereby hope to have clarified all concerns and would like to thank the reviewers for their comments, which we believe supported the credibility of the results, made the paper more understandable, and will help the reader to better navigate through the text.

Best regards,

Simone Lederer
